# Effects of cholesterol-lowering probiotics on non-alcoholic fatty liver disease in FXR gene knockout mice

**DOI:** 10.3389/fnut.2023.1121203

**Published:** 2023-07-20

**Authors:** Minghua Yang, Haoyang Wang, Ihtisham Bukhari, Ye Zhao, Huang Huang, Yong Yu, Xiangdong Sun, Yang Mi, Lu Mei, Pengyuan Zheng

**Affiliations:** ^1^Department of Gastroenterology, The Fifth Affiliated Hospital of Zhengzhou University, Zhengzhou, China; ^2^Henan Key Laboratory for Helicobacter Pylori & Microbiota and GI Cancer, Marshall Medical Research Center, The Fifth Affiliated Hospital of Zhengzhou University, Zhengzhou, China

**Keywords:** NAFLD, FXR, gut microbiota, probiotics, bile acids

## Abstract

**Background/aims:**

Some studies showed that probiotics could improve the composition and structure of gut microbiota. Changes in the gut microbiota may alter bile acid (BAs) composition and kinetics, improving non-alcoholic fatty liver disease (NAFLD). However, it still needs to be clarified how probiotics improve both the metabolism of BAs and NAFLD. This study aimed to reveal the regulatory mechanisms of cholesterol-lowering (CL) probiotics on NAFLD from aspects involved in BA metabolism in FXR gene knockout (FXR^−/−^) mice.

**Methods:**

FXR^−/−^ male mice were randomly divided into three groups based on different interventions for 16 weeks, including normal diet (ND), high-fat diet (HFD), and probiotic intervention in the HFD (HFD+P) group. 16s rDNA sequencing and ultrahigh performance liquid chromatography-tandem mass spectrometry (UHPLC-MS/MS) were utilized to analyze the changes in gut microbiota and fecal bile acids in mice.

**Results:**

We found that the intervention of the CL probiotics improved liver lipid deposition and function in HFD-induced NAFLD mice by decreasing the levels of total cholesterol (TC; *p* = 0.002) and triglyceride (TG; *p* = 0.001) in serum, as well as suppressing liver inflammation, such as interleukin-1 beta (IL-1β; *p* = 0.002) and tumor necrosis factor-alpha (TNF-α; *p* < 0.0001). 16S rDNA sequencing and metabolomic analyses showed that probiotics effectively reduced the abundance of harmful gut microbiota, such as Firmicutes (*p* = 0.005), while concomitantly increasing the abundance of beneficial gut microbiota in NAFLD mice, such as Actinobacteriota (*p* = 0.378), to improve NAFLD. Compared with the ND group, consuming an HFD elevated the levels of total BAs (*p* = 0.0002), primary BAs (*p* = 0.017), and secondary BAs (*p* = 0.0001) in mice feces, while the intervention with probiotics significantly reduced the increase in the levels of fecal total bile acids (*p* = 0.013) and secondary bile acids (*p* = 0.017) induced by HFD.

**Conclusion:**

The CL probiotics were found to improve liver function, restore microbiota balance, correct an abnormal change in the composition and content of fecal bile acids, and repair the damaged intestinal mucosal barrier in mice with NAFLD, ultimately ameliorating the condition. These results suggested that CL probiotics may be a promising and health-friendly treatment option for NAFLD.

## Introduction

Non-alcoholic fatty liver disease (NAFLD), a condition in which excess fat accumulates in the liver of individuals who consume little or no alcohol, is becoming a significant public health concern globally (1). It affects about 25% of the world's population, and the incidence rates are rising at an alarming rate due to the increasing prevalence of risk factors such as obesity, type 2 diabetes, and metabolic syndrome, contributing to the global increase in chronic liver disease cases (2). NAFLD includes a wide range of pathological liver changes from simple degeneration to non-alcoholic steatohepatitis, fibrosis, and eventually cirrhosis (3). Although NAFLD has been thoroughly studied for decades, its pathogenesis is still poorly understood, and treatment options are limited (4). Studies showed that insulin resistance, genetic factors, and lifestyle choices, such as poor diet and a lack of physical activity, play a significant role in its development (5). However, the exact mechanisms that lead to the accumulation of fat in the liver and the progression of the disease are complex and multifactorial (6). Early detection and management of NAFLD are crucial for preventing its progression and improving patient outcomes (7). Lifestyle modifications, including a balanced diet, regular exercise, and weight loss, are the first-line treatment options for NAFLD (8). Pharmacotherapy may sometimes be necessary to manage associated comorbidities (9). Despite limited therapeutic options, ongoing research aims to identify new targets and treatments to fully address this growing public health concern.

Under normal physiological conditions, an intestinal mucosal barrier protects against harmful intestinal substances and prevents infections by translocating them to the liver (10). The paracellular permeability increased, and the structure of tight junction proteins was destructed due to declined expression of Claudin-1, Claudin-3, and ZO-1 (11). Accordingly, bacteria can pass through the intestinal wall, leading to injury and inflammation in the liver (12). In addition, weak intestinal mucosal barrier function can be driven by liver-derived inflammatory cytokines that destroy the intestinal tight junction (13). The functional association between the gut and liver can ameliorate the pathogenesis and suppress the progression of NAFLD (14).

The gut microbiota is regarded as the largest microbial community in our body and plays a vital role in the homeostasis of the host microenvironment (15). The alterations in gut microbiota are highly associated with obesity and NAFLD, e.g., reduced Firmicutes-to-Bacteroidota (F/B) ratio and microbial diversity (16). Moreover, dysbiosis in gut microbiota can further change liver functions by translocating the bacteria-derived proinflammatory substance across the gut–liver axis, resulting in disturbed lipid metabolism that contributes to the onset or progression of NAFLD (14, 17). Recent studies have highlighted some critical approaches for modulating the gut microbiota, such as diet modification, prebiotics, and probiotic supplementation (18, 19). These methods promote beneficial microbial strains, reducing pathogenic gut bacteria composition, improving gut barrier function and immune regulation, and better managing metabolic diseases (20). Further studies are required to determine optimal treatment strategies for long-term gut microbiome modulation and beneficial outcomes for patients with NAFLD.

Bile acids (BAs) are discharged from the liver, stored in the gallbladder, and secreted into the gastrointestinal tract, which plays an essential role in energy metabolism and promoting the absorption of nutrients (21). Approximately 5–10% of BAs are metabolized by enzymes in the ileum and excreted in the stool, with the remainder being reabsorbed in the ileum (22). FXR is a vital receptor of BAs and while increasing its excretion to maintain the BA homeostasis (23). It has been confirmed as a promising pharmacological treatment for high-fat diet (HFD)-induced hyperlipidemia since activated FXR regulates hepatic BA synthesis (24, 25). Notably, NAFLD patients' BA composition differed significantly from healthy subjects (26). In parallel, there is a close interaction between gut microbiota and bile acids. The BA metabolism is regulated by gut microbiota, and primary BAs are converted into secondary BAs under the gut microbiota (27, 28). The BAs also affect the community structure of gut microbiota (29). We thus hypothesize that there is a strong association between NAFLD disease severity and dysregulation of BA homeostasis.

Some studies have confirmed that probiotics could decrease the level of cholesterol and balance the disturbed homeostasis in human beings (30, 31). Previously, we showed that cholesterol-lowering (CL) probiotics, DM9054 and 86066, not only regulate lipid metabolism and reduce the release of inflammatory cytokines but also restore intestinal barrier function and maintain the stability of the intestinal microbiota, which provides a potential therapy for NAFLD (32, 33). However, despite these promising findings, the specific mechanism whereby probiotics achieve these beneficial effects still needs to be defined. Accordingly, we aimed to assess the impact of CL probiotics on BAs composition and the gut microbiota of FXR gene knockout (FXR^−/−^) mice with HFD-induced NAFLD.

## Materials and methods

### Probiotic strains and animals

CL probiotics *Lactobacillus rhamnosus* DM9054 and *Lactobacillus plantarum* 86066 were screened in our previous study (32). FXR^−/−^ mice were purchased from Saiye (Suzhou) Biotechnology Co., LTD. [Experimental Animal Production License No. SCXK (Su) 2018-0003] and then crossbred with C57BL/6 mice repeatedly. We obtained mice with the FXR gene completely knocked out. Four-week-old male FXR^−/−^ mice were raised in specific pathogen-free (SPF) environments. The Animal Ethics Committee of the Fifth Affiliated Hospital of Zhengzhou University approved the experimental and sampling procedures on mice (KY2021039).

The male FXR^−/−^ mice were randomly separated into three groups: (1) ND group: mice were fed on ND for 16 weeks and administered normal saline (10 ml/kg) intragastrically daily; (2) HFD group: mice were fed on 45% HFD continuously for 16 weeks and administered normal saline (10 ml/kg) intragastrically; (3) HFD+P group: mice were fed on 45% HFD continuously for 16 weeks and administered probiotics (1 × 10^10^ CFU per ml in 0.9% NaCl, 1 ml/day) intragastrically. We recorded the mice's avoirdupois weight at the end of the intervention.

### Biochemical analysis

Mice serum samples were collected to determine the levels of total bile acids (TBA), triglyceride (TG), total cholesterol (TC), low-density lipoprotein cholesterol (LDL-C), high-density lipoprotein cholesterol (HDL-C), aspartate transaminase (AST), and alanine aminotransferase (ALT) by colorimetric kits (Shenzhen Kubel Biotechnology Co., LTD, Shenzhen, China).

For the insulin tolerance test, mice were transferred to clean cages and fasted for 8 h. Then, they were injected intraperitoneally with 0.1 U/ml of insulin solution. Venous blood samples from the tails of mice were obtained at 0, 15, 30, 60, 90, and 120 min before and after insulin injection to check blood glucose with a glucose meter. In addition, the same method of disposal and detection was applied to the glucose tolerance test using glucose solution (20%, sterile).

### Measurement of inflammatory cytokines

An ELISA kit (Nanjing Jiancheng Bioengineering Institute, Nanjing, China) was used to determine the concentration of inflammatory cytokines in the tissue samples: tumor necrosis factor-alpha (TNF-α), interleukin-1 beta (IL-1β), and interleukin-6 (IL-6) in the liver and ileum homogenate of mice.

### Histopathological examination

The specimens were obtained from the liver and intestine of mice and then fixed using 4% paraformaldehyde. We used paraffin to embed fixed specimens and cut them into approximately 5-mm sections to assess the effect of CL probiotics on the liver histology and morphology of the NAFLD mice.

### Quantitative real-time PCR

The liver and ileum tissues from mice were used for mRNA quantification. Total RNA was taken from those tissues using TRIzol reagent (Invitrogen Company, USA) and then reverse transcribed into cDNA using a ReverTra Ace^®^ qPCR RT Kit (TOYOBO, Japan). We used quantitative real-time PCR (qRT-PCR) to perform the gene amplification. The specific primer series are presented in Table 1. In addition, we performed the 2^−ΔΔCT^ method to determine the expression level of the targeted genes.

**Table 1:** Primer sequences for qRT-PCR.

	**Forward primer (5′-3′)**	**Reverse primer (5′-3′)**
GAPDH	GCTGAGTATGTCGTGGAG	TCTTCTGAGTGGCAGTGAT
TNF-α	GAAAAGCAAGCAACCAGCCA	CGGATCATGCTTTCCGTGC TC
TLR4	TCAGCAAAGTCCCTGATGACA	CCTGGGGAAAAACTCTGGATAG
SHP	CGATCCTCTTCAACCCAGATG	AGGGCTCCAAGACTTCACACA
IL-1β	CAAACTGATGAAGCTCGTCA	TCTCCTTGAGCGCTCACGAA
IL-6	AGTTGCCTTCTTGGGACTGA	TCCACGATTTCCCAGAGAAC
CYP7A1	GCAAAACCTCCAATCTGTCAT	GCTTCAAACATCACTCGGTAAC
SR-B1	ATAAAGCCTCTGGCCACCTG	CATGAAGGGTGCCCACATCT

qRT-PCR, quantitative real-time PCR.

### Western blot

We extracted total protein from the liver and intestine of mice. We used sodium dodecyl sulfate-polyacrylamide gel electrophoresis (SDS-PAGE) to separate equal amounts of proteins, and then the proteins were transferred onto nitrocellulose membranes. BCA protein quantitative kits (Biyuntian Biotechnology Co., LTD, Shanghai, China) were used to measure tissue-homogenized protein concentration. The membranes were incubated with the antibody overnight at 4°C. Subsequently, after washing with PBS, the membranes were incubated with a secondary antibody at 25°C for 1 h. We used an enhanced chemiluminescence (ECL) detection system to detect the protein bands.

### 16S rDNA gene sequencing and analysis

The feces samples of mice were collected and sent to Meggie Biomedical Technology Co., LTD (Shanghai, China) for 16S rDNA sequencing. The AxyPrep DNA Gel Extraction Kit was used to extract the total bacterial DNA of mice fecal samples. The V3–V4 variable region of the 16S rRNA gene was amplified. The Illumina MiSeq sequencing amplification library was constructed using the bacterial primers 338F (5′-ACTCCTACGGGAGGCAGCAG-3′) and 806R (5′-GGACTACHVGGGTWTCTAAT−3′), targeting the V3–V4 hypervariable region of bacterial 16S rRNA gene. Then, the Illumina MiSeq platform was conducted to sequence amplicons based on the standard protocols; then operational taxonomic unit (OTU) clustering was carried out. We used these indicators to evaluate alpha diversity and beta diversity. The community structure was statistically analyzed based on the classification information at the phylum and genus levels.

### Determination of fecal bile acid levels

The BA content in mice fecal samples was determined using ultrahigh-performance liquid chromatography-tandem mass spectrometry (UHPLC-MS/MS) from Meggie Biomedical Technology Co., LTD (Shanghai, China). We mixed 400 μl of extract solution (methanol:water, 4: 1) and a 50-mg aliquot of every sample, and then the mixture was ground for 6 min (−10°C, 50 Hz) by the cold grinding machine and sonicated for half an hour via a refrigerating circulator, then centrifuged at 4°C and 13,000 rpm for 15 min and incubated at −20°C for 30 min. The UHPLC-MS/MS analysis was performed after supernatants were transferred to LC-MS vials using a Waters BEH C18 column (100 × 2.1 mm, 1.7 μm, Waters).

### Statistical analysis

Raw data were assessed as mean ± standard deviation (SD). The multiple group comparisons were analyzed using GraphPad Prism 8.4 software with a one-way ANOVA. The results were taken as a notable difference with *p* < 0.05.

## Results

### CL probiotics improved the metabolic parameters of NAFLD mice

**Figure 1:**
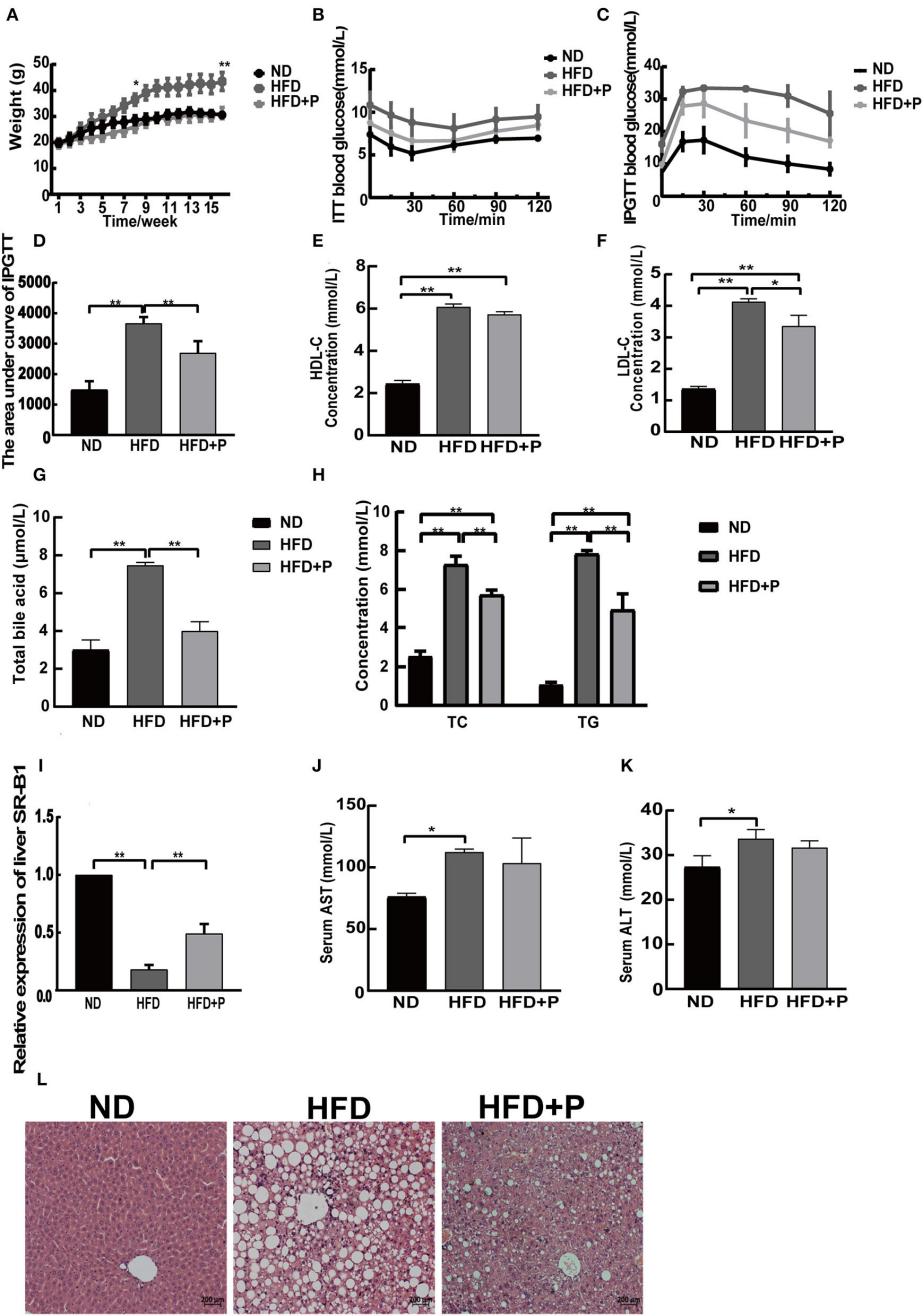
Effects of the cholesterol-lowering probiotics on liver steatosis and function in HFD mice. **(A)** Body weight curve; **(B)** ITT blood glucose; **(C)** IPGTT blood glucose; **(D)** the area under the curve of IPGTT; **(E)** serum HDL-C level; **(F)** serum LDL-C level; **(G)** total bile acid; **(H)** serum TC and TG levels; **(I)** relative expression of liver SR-B1; **(J)** serum AST level; **(K)** serum ALT level; **(L)** the HE-stained liver section. All data are given as the mean ± SD (*n* = 3). *p < 0.05; **p < 0.01. ND, normal diet; HFD, high-fat diet; HFD+P, probiotics intervention in HFD; ITT, insulin tolerance test; IPGTT, intraperitoneal glucose tolerance test; HDL-C, high-density lipoprotein cholesterol; LDL-C, low-density lipoprotein cholesterol; TC, total cholesterol; TG, triglyceride; SR-B1, scavenger receptor class B type1; AST, aspartate aminotransferase; ALT, alanine aminotransferase.

In comparison to the ND group (28.54 ± 1.72), the body weights of HFD mice (36.45 ± 2.19) elevated remarkably from the eighth week (*p* = 0.015). After 16 weeks of feeding, there was a significant difference in the body weight of mice between the HFD+P group (31.28 ± 2.15) and the HFD group (43.45 ± 3.49), with the former demonstrating a notably lower body weight compared with the latter (*p* = 0.0002; Figure 1A). The CL probiotics potentially reduced the body weight of HFD mice. Meanwhile, we found that the insulin sensitivity of HFD mice (33.20 ± 0.40) was increased by CL probiotics as demonstrated by the intraperitoneal glucose tolerance test (IPGTT) levels in the HFD+P group (23.33 ± 4.989) of mice from the 60th minute (*p* = 0.005; Figures 1B–D).

The biochemical parameters in the serum of mice showed that the levels of serum LDL-C (4.127 ± 0.96), HDL-C (6.077 ± 0.138), TC (7.280 ± 0.437), TG (7.837 ± 0.172), and total BA (7.467 ± 0.152) were upregulated in HFD mice compared with the ND group (1.357 ± 0.083, 2.433 ± 0.162, 2.540 ± 0.266, 1.077 ± 0.126, and 3.000 ± 0.529, respectively; *p* < 0.0001, all). At the same time, probiotic intervention efficiently reduced LDL-C (*p* = 0.010), TC (*p* = 0.002), TG (*p* = 0.0009), and total BA (*p* = 0.0001) in the HFD group, except for the level of serum HDL-C (*p* = 0.0540; the level of LDL-C, TC, TG, total BA, and HDL-C in the HFD+P group was 3.353 ± 0.344, 5.713 ± 0.251, 4.943 ± 0.827, 3.990 ± 0.496, and 5.723 ± 0.128, respectively; Figures 1E–H). Then, the relative expression level of hepatic scavenger receptor class B type1 (SR-B1) was examined (Figure 1I). The results showed that HFD reduced the hepatic SR-B1 expression in the HFD group in comparison to the ND group (*p* < 0.0001); however, it was recovered in the HFD+P group after the intervention of probiotics (*p* < 0.0001). In addition, the serum alanine aminotransferase (ALT) and aspartate transaminase (AST) levels in the ND group were 27.33 ± 2.517 and 76.00 ± 3.000, respectively. In contrast, in HFD mice, the corresponding levels increased to 33.67 ± 2.082 and 112.3 ± 2.517, which showed a certain degree of increase compared with those of the ND group mice (*p* = 0.022 and *p* = 0.023, respectively). Notably, the levels of ALT and AST in the HFD+P group were found to be 31.67±1.528 and 103.3±20.50, respectively, which showed a reduced trend compared with HFD mice, but there was no statistical significance (*p* = 0.507 and *p* = 0.651, respectively; Figures 1J, K).

H&E staining showed that the percentage of cells with lipid droplets and lipid droplet size was reduced under the treatment of probiotics; moreover, the fat accumulation in the liver of HFD mice was inhibited by the probiotic treatment (Figure 1L). Our results suggested that the intervention of the CL probiotics improved liver lipid deposition and function in mice with HFD-induced NAFLD.

### CL probiotics suppress inflammation in mice fed with HFD

**Figure 2:**
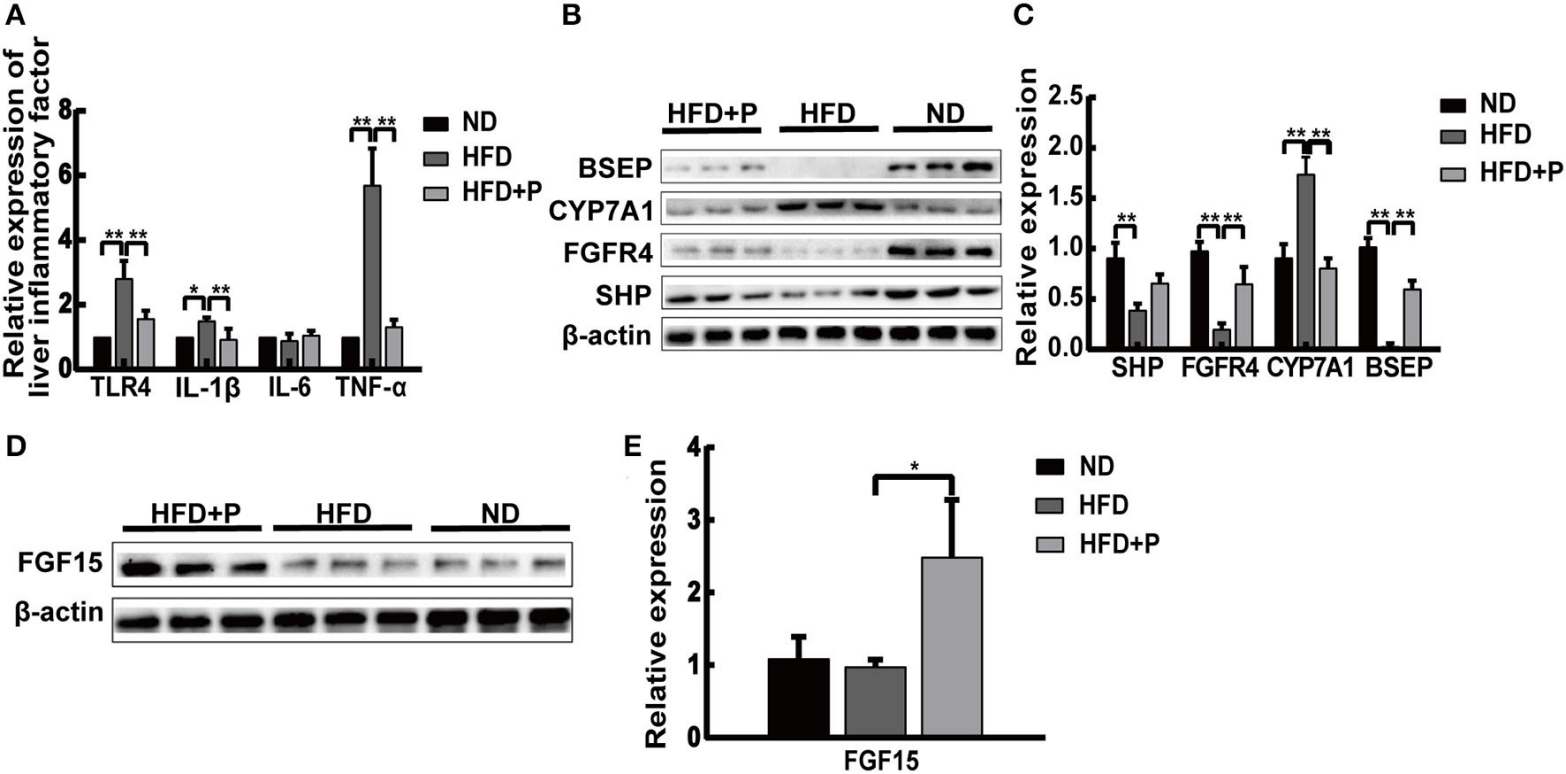
Cholesterol-lowering probiotics suppress inflammation in mice fed with HFD. **(A)** The mRNA expression levels of TLR4, TNF-α, IL-6, and IL-1β were detected by real-time PCR. **(B)** The protein expression of BSEP, CYP7A1, FGFR4, and SHP was detected by Western blotting. **(C)** Relative mRNA expression levels of genes and protein involved in cholesterol metabolism and bile acid synthesis. **(D)** Western blot analysis of intestinal FGF15 expression. **(E)** Bar graphs for the plots of FGF15. All data are given as the mean ± SD (*n* = 6). *p < 0.05; **p < 0.01. ND, normal diet; HFD, high-fat diet; HFD+P, probiotics intervention in HFD; TLR4, toll-like receptor 4; TNF-α, tumor necrosis factor-alpha; IL-6, interleukin-6; IL-1β, interleukin-1 beta; BSEP, bile salt export pump; CYP7A1, cholesterol 7α-hydroxylase; FGFR4, fibroblast growth factor receptor 4; SHP, short heterodimer partner; FGF15, fibroblast growth factor 15.

An increased level of toll-like receptor 4 (TLR4) in the liver was detected in the HFD mice (*p* = 0.001; Figure 2A), indicating that lipopolysaccharide (LPS) from the intestinal microbiota has transferred into the blood, leading to hyper-expression of TLR4 and other inflammatory responses. The levels of IL-1β and TNF-α were also increased in HFD mice (*p* = 0.016 and *p* < 0.0001, respectively; Figure 2A), while probiotic treatment reduced the levels of these cytokines (*p* = 0.001 and *p* < 0.0001, respectively). Our results indicated that the CL probiotics attenuated inflammatory response in mice with HFD-induced NAFLD.

The Western blot results showed that basal expressions of fibroblast growth factor receptor 4 (FGFR4), short heterodimer partner (SHP), and bile salt export pump (BSEP) in the liver of HFD mice were lower than those of the ND group (*p* < 0.0001, *p* = 0.001, and *p* < 0.0001, respectively). The levels of FGFR4 and BSEP were upregulated under the treatment of CL probiotics (*p* = 0.0058 and *p* < 0.0001, respectively; Figures 2B, C). The expression levels of cholesterol 7α-hydroxylase (CYP7A1) in HFD mice were higher than in the ND group (*p* < 0.0001). CL probiotic intervention markedly inhibited the level of CYP7A1 (*p* < 0.0001). The expression level of intestinal fibroblast growth factor 15 (FGF15) was augmented significantly in the HFD+P group in comparison with HFD mice (*p* = 0.026; Figures 2D, E).

### The intervention of the CL probiotics restored intestinal barrier function in HFD mice

**Figure 3:**
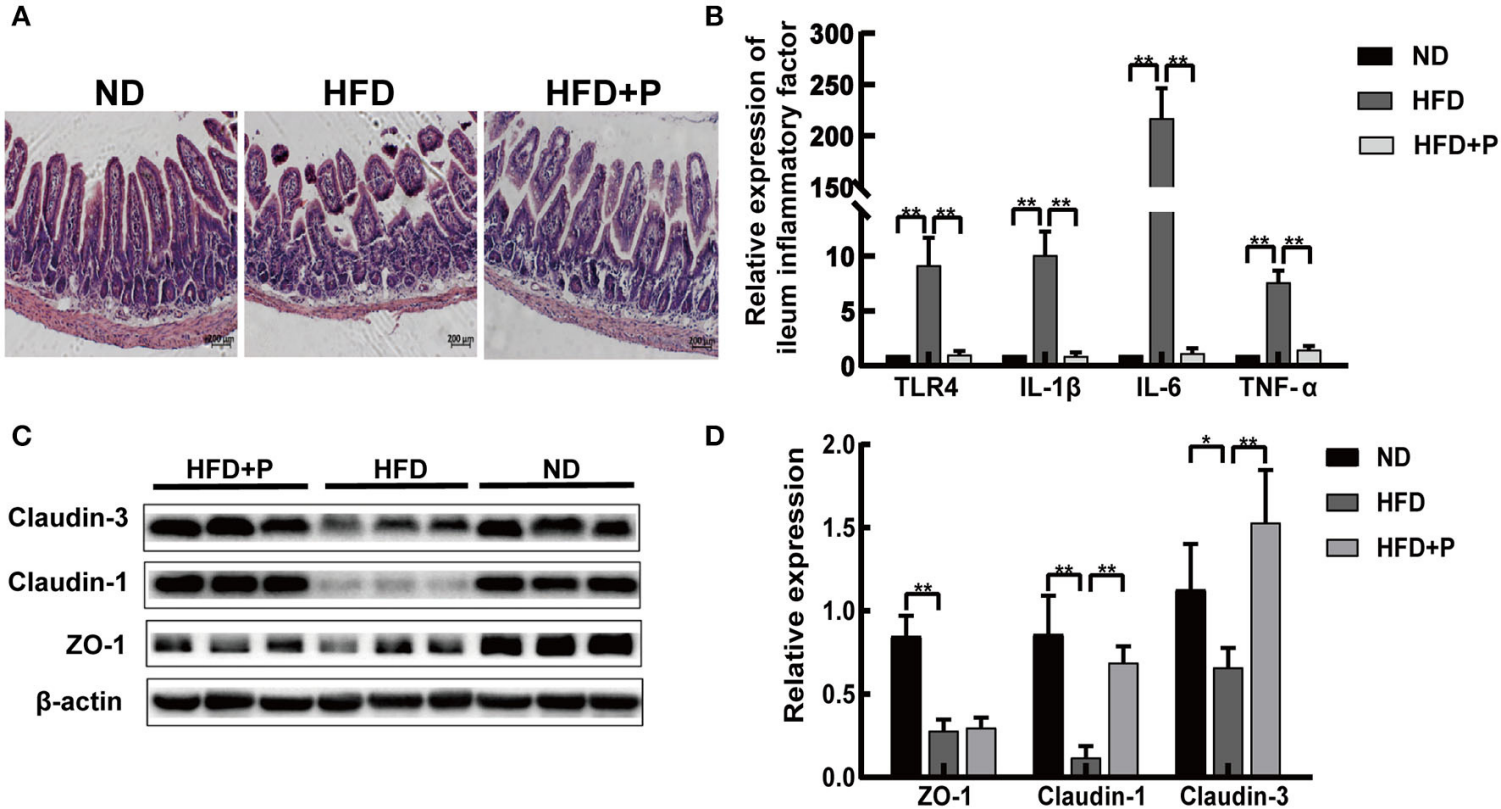
Cholesterol-lowering probiotics restored the function of the intestinal barrier in HFD mice. **(A)** H&E staining of intestinal tissue sections. **(B)** qRT-PCR detected relative gene expression of TLR4, IL-1β, IL-6, and TNF-α in the ileum. **(C)** Western blot analysis of ZO-1, Claudin-1, and Claudin-3 expression in the intestinal tissue. **(D)** Bar graphs for the plots of ZO-1, Claudin-1, and Claudin-3. All data are given as the mean ± SD (*n* = 6). *p < 0.05; **p < 0.01. ND, normal diet; HFD, high-fat diet; HFD+P, probiotics intervention in HFD; H&E, Hematoxylin and Eosin staining; qRT-PCR, quantitative real-time PCR; TLR4, toll-like receptor 4; IL-1β, interleukin-1 beta; IL-6, interleukin 6; TNF-α, tumor necrosis factor alpha; ZO-1, tight junction protein.

The ileum villi base in the HFD mice was broken, especially its arrangement (Figure 3A). After the intervention of the CL probiotics, we observed that the ileum structure returned to normal (Figure 3A). The mRNA expression level of ileum inflammatory factors (IL-1β, IL-6, and TNF-α) in HFD mice was higher than in mice with ND (*p* < 0.0001, all) while reduced in the probiotics-treated group (*p* < 0.0001, all; Figure 3B). Our results indicated that compared with the ND group, the expression of intestinal Claudin-1, Claudin-3, and tight junction protein 1 (ZO-1) reflecst the permeability of the intestinal barrier; these proteins decreased in the HFD group, suggesting damage to the integrity of the intestinal mucosal barrier (*p* = 0.002, *p* = 0.032, and *p* = 0.0005, respectively; Figures 3C, D). Moreover, Claudin-1 and Claudin-3 proteins were more highly expressed in the HFD+P group (*p* < 0.0001 and *p* = 0.0048, respectively; Figure 3D). These data revealed that CL probiotics could protect the intestinal barrier of the NAFLD mice.

### CL probiotics modulated intestinal dysbiosis in mice with HFD-induced NAFLD

#### CL probiotics increased the diversity of intestinal microbiota in NAFLD mice

**Figure 4:**
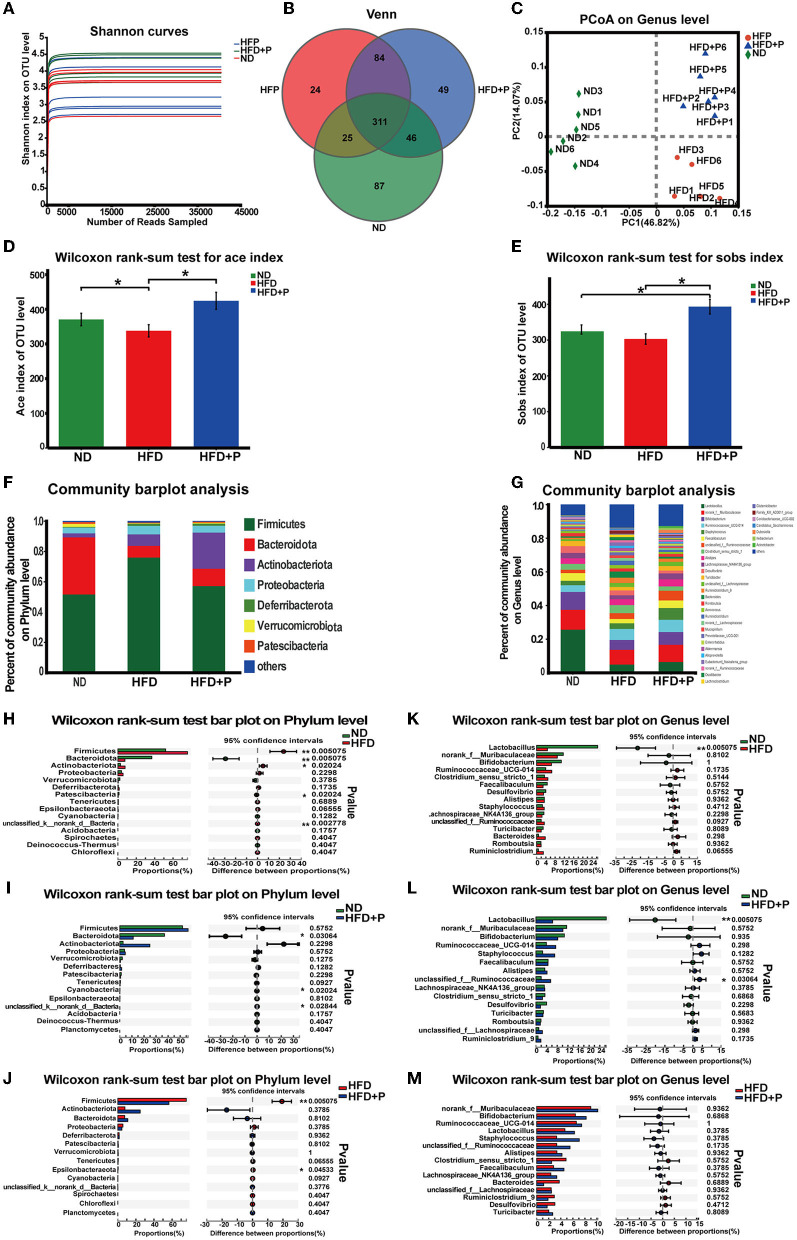
Effects of the cholesterol-lowering probiotics on the structure of gut microbiota. **(A)** The Shannon index represents the diversity of samples. **(B)** Venn diagram analyzes the diversity differences between different groups. **(C)** The PCoA score plot based on unweighted β diversity analysis. **(D)** The Sobs index and **(E)** the Ace index represents the α diversity of samples. **(F)** Relative abundance of microbiota at the phylum level. **(G)** Relative abundance of microbiota at the genus level; **(H–J)** Differences at the phylum level between the two groups were tested. **(K–M)** Differences at the genus level between the two groups were tested. All data are given as the mean ± SD (*n* = 6). *p < 0.05; **p < 0.01. ND, normal diet; HFD, high-fat diet; HFD+P, probiotics intervention in HFD.

The microbial diversity in the sample was reflected by enough sequencing data (Figure 4A). The Venn chart revealed that the variety of microorganisms in the HFD mice decreased compared with the ND group. However, in the HFD+P group, it was reversed (Figure 4B). Furthermore, intestinal microbe composition in the ND, HFD, and HFD+P groups was distinguished (Figure 4C). Sobs and Ace indexes indicated the richness and evenness of species and showed species diversity in a community. There was a significant separation trend among the ND group, HFD group, and HFD+P group, reflecting that HFD decreased gut microbiota diversity compared with the ND group (*p* = 0.030); at the same time, CL probiotics improved gut microbiota diversity compared with the HFD mice (*p* = 0.02; Figures 4D, E).

#### CL probiotics alleviated intestinal dysbiosis in mice with HFD-induced NAFLD

At the phylum level, our research revealed that compared with the ND group, the abundance of Firmicutes and Proteobacteria was upregulated in HFD mice. In contrast, the abundance of Bacteroidota was less (Figure 4F). The results of the Wilcoxon rank-sum test showed that the HFD significantly increased the abundance of Firmicutes (*p* = 0.005). In contrast, the abundance of Bacteroidota decreased (*p* = 0.005; Figure 4H). In addition, the supplementation of CL probiotics augmented the quantity of Actinobacteriota. It also reduced the proportion of Proteobacteria and Firmicutes, which differed significantly from the results of the mice of the HFD group (Figure 4F). Compared with the ND group, the abundance of Bacteroidota in the HFD+P group was significantly lowered (*p* = 0.030; Figure 4I). The Wilcoxon rank-sum test indicated that compared with the HFD group, the intervention of the CL probiotics reduced the number of Firmicutes in mice with HFD (*p* = 0.005; Figure 4J).

At the genus level, we observed a significant enrichment of Ruminococcaceae-UCG-014 and depletion of *Lactobacillus*, an unknown genus of *Muribaculaceae, Bifidobacterium*, and *Faecalibaculum* in the HFD mice (Figure 4G). A notable difference was observed in the abundance of *Lactobacillus* between mice fed with ND and HFD (*p* = 0.005; Figure 4K). Moreover, the abundance of *Lactobacillus* was lowered in the HFD+P group, compared with the ND group (*p* = 0.005; Figure 4L). Compared with HFD mice, there was an increased trend in the quantity of several bacterial genera in the HFD+P group, including *Lactobacillus*, the unknown genus of *Muribaculaceae, Bifidobacterium, Ruminococcaceae-UCG-014*, and *Faecalibaculum* (Figure 4M). Our results suggested that CL probiotics could modulate HFD-induced gut microbiota dysbiosis in mice.

#### Spearman's correlations between gut microbial genera and NAFLD-associated metabolic parameters

**Figure 5:**
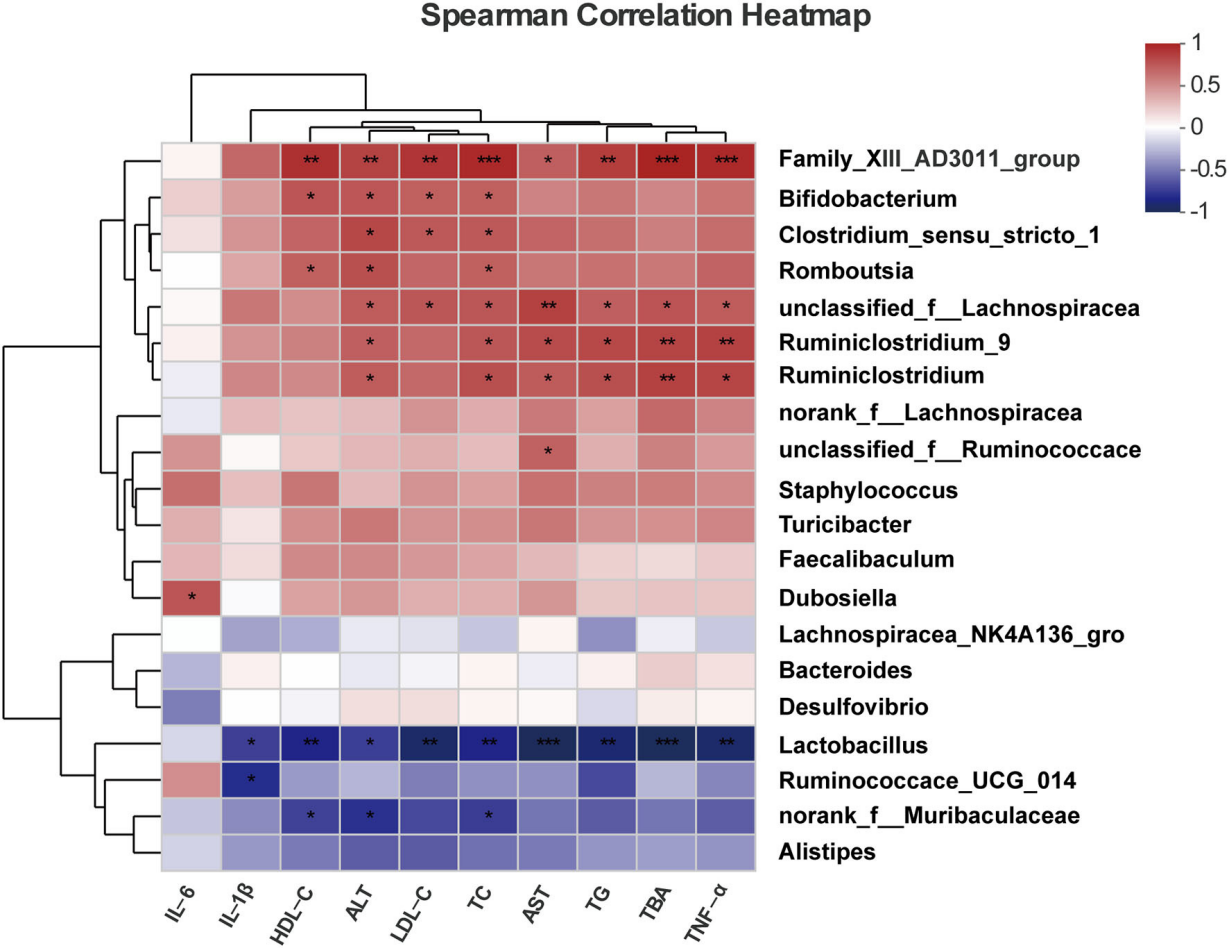
Spearman's correlations between the gut microbial community at the genus level and vital metabolic parameters linked to NAFLD. All data are given as the mean ± SD (*n* = 6). *p < 0.05; **p < 0.01; ***p < 0.001.

Spearman's correlation heatmap showed the associations of critical metabolic parameters related to NAFLD, and these parameters were regulated by the treatment of probiotics and the key bacterial groups (Figure 5). Our results showed that bacteria genera enriched in the ND and HFD+P groups, such as *Lactobacillus*, negatively correlated with NAFLD-related metabolic parameters and liver inflammatory factors, such as the levels of serum TC (*r* = −0.8, *p* = 0.009), TG (*r* = −0.85, *p* = 0.003), AST (*r* = −0.933, *p* = 0.0002 < 0.001), ALT (*r* = −0.692, *p* = 0.038), HDL-C (*r* = −0.8 *p* = 0.009), LDL-C (*r* = −0.866, *p* = 0.002), TBA (*r* = −0.916, *p* = 0.0005), IL-1β (*r* = −0.695, *p* = 0.037), and TNF-α (*r* = −0.864, *p* = 0.002). One sub-group of *Muribaculaceae* was also negatively correlated with some NAFLD-associated metabolic parameters, such as HDL-C (*r* = −0.683, *p* = 0.042), ALT (*r* = −0.751, *p* = 0.01), and TC (*r* = −0.7, *p* = 0.035). While the bacteria increased in the HFD group such as *Clostridium-sensu-stricto-1* and *Ruminiclostridium*-9 were positively correlated with ALT (*r* = 0.789 and *p* = 0.0113, *r* = 0.716 and *p* = 0.0298, respectively) and TC (*r* = 0.711 and *p* = 0.0314, *r* = 0.71 and *p* = 0.0298, respectively) levels in mice. These findings suggest that modulating specific bacterial taxa in the gut microbiota may profoundly impact metabolic health.

### Effect of CL probiotics on the fecal BA composition of mice with HFD-induced DAFLD contents

**Figure 6:**
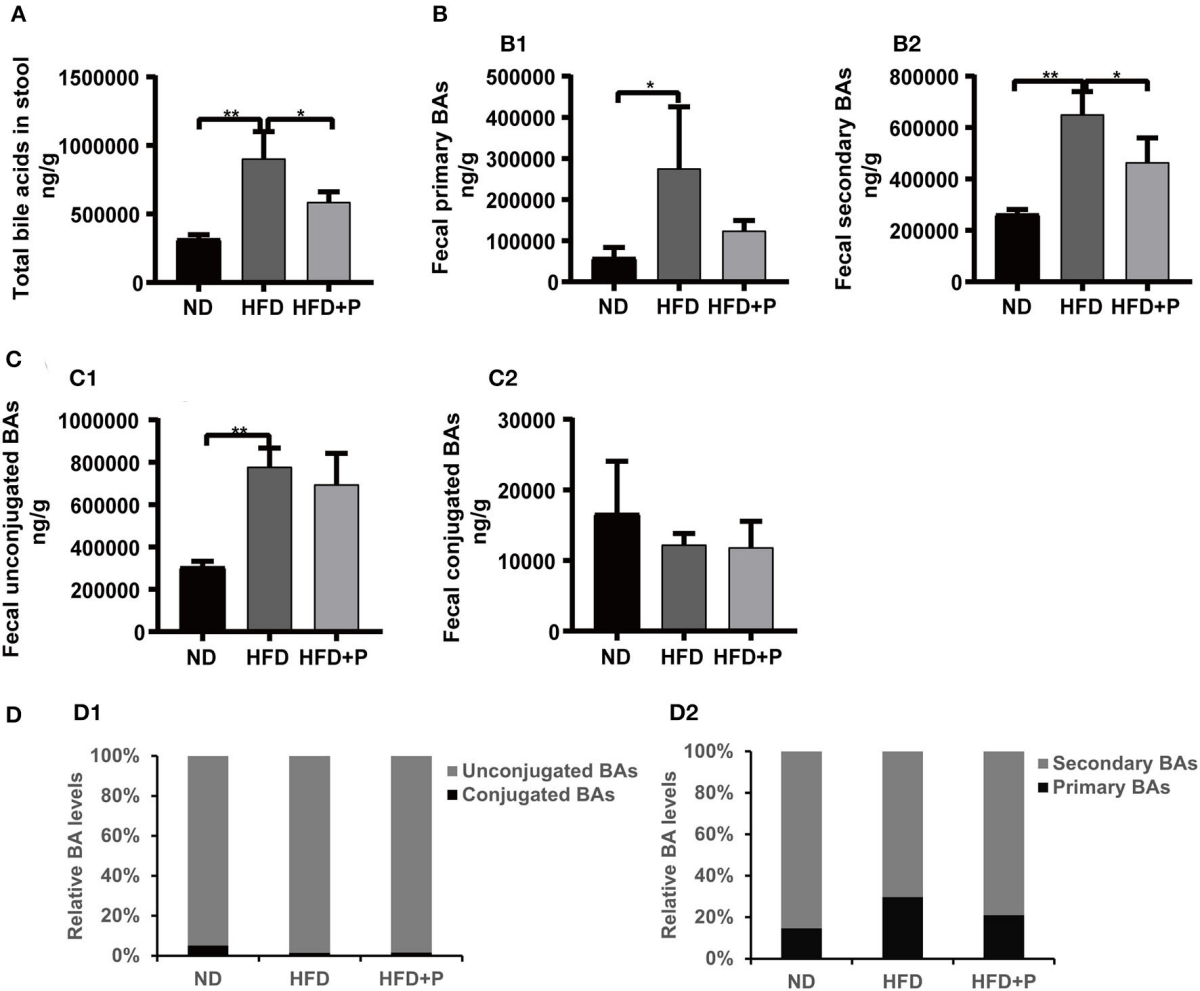
Effect of cholesterol-lowering probiotics on the variation of fecal BA contents. **(A)** Total fecal BA levels. **(B1)** Primary fecal BAs and **(B2)** Secondary BAs. **(C1)** Fecal unconjugated BAs and **(C2)** Conjugated Bas. **(D1)** Relative primary BA and secondary BA levels (% of the total BA). **(D2)** Relative unconjugated BA and conjugated BA levels (% of the total BA). All data are given as the mean ± SD (*n* = 4). *p < 0.05; **p < 0.01. ND, normal diet; HFD, high-fat diet; HFD+P, probiotics intervention in HFD; BAs, bile acids.

**Figure 7:**
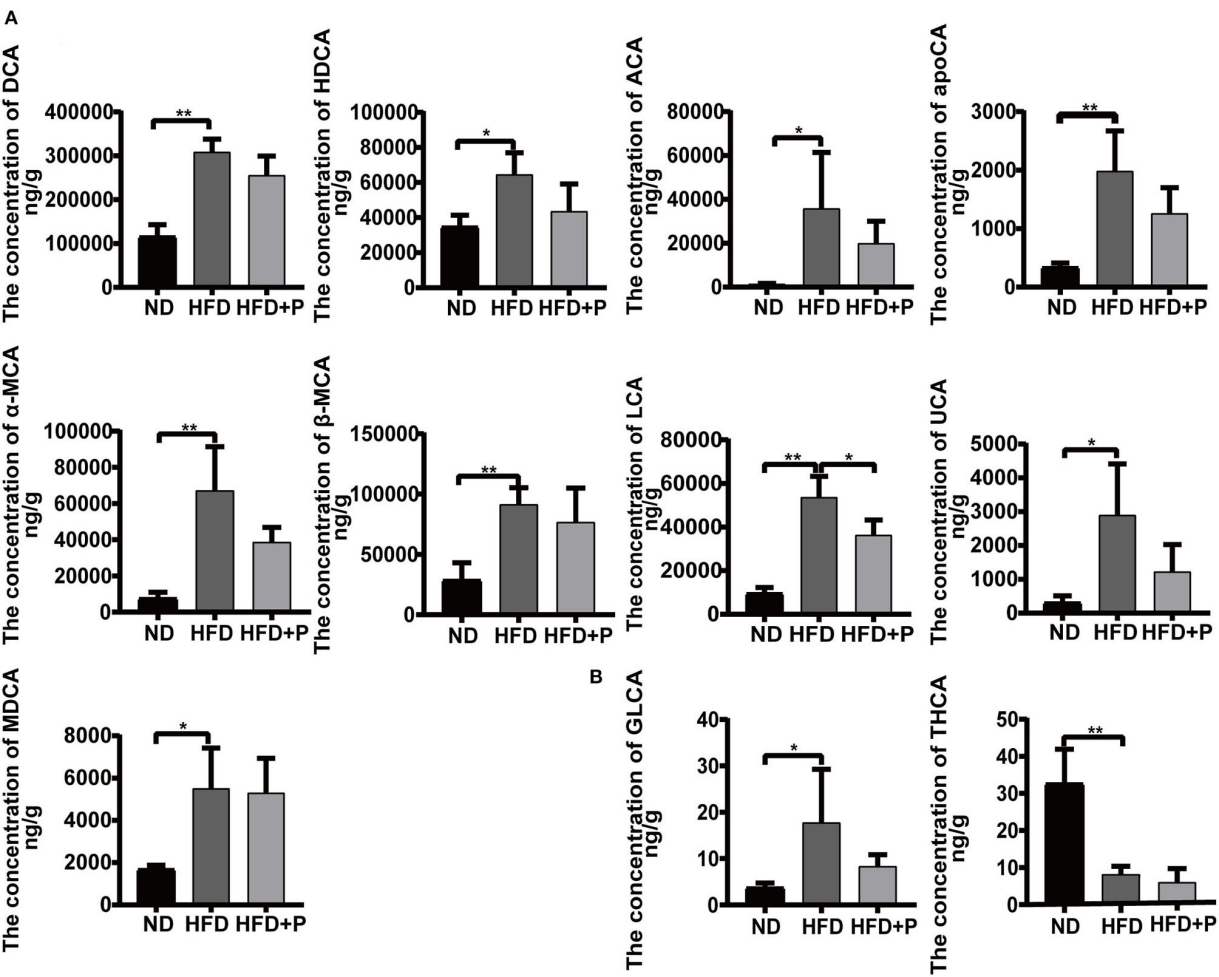
Effect of cholesterol-lowering probiotics on fecal bile acid monomers. **(A)** Unconjugated bile acid monomers. **(B)** Conjugated bile acid monomers. ACA, allocholic acid; DCA, deoxycholic acid; apoCA, apocholic acid; HDCA, hyodeoxycholic acid; α-MCA, α-muricholic acid; β-MCA, β-muricholic acid; LCA, lithocholic acid; MDCA, murideoxycholic acid; UCA, ursocholic acid; GLCA, glycolithocholic acid; THCA, taurohyocholic acid. All data are given as the mean ± SD (*n* = 4). *p < 0.05; **p < 0.01. ND, normal diet; HFD, high-fat diet; HFD+P, probiotics intervention in HFP.

The current study showed that alterations in the composition and content of bile acids were a valuable indicator of metabolic disorder status in mice (Figures 6, 7, and Supplementary Figure 1). The total BAs (TBAs) content in mice feces showed significant differences among the three groups. Specifically, in the ND group, the TBAs level was 317,863 ± 30,962; in the HFD group, it dramatically increased to 903,763 ± 197,675, significantly higher than that in the ND group (*p* = 0.0002). In contrast, in the HFD+P group, the TBAs level was 587,369 ± 74,196, indicating a certain degree of decline. Notably, the probiotics' intervention significantly reduced the levels of TBAs in mice feces when compared with the HFD group (*p* = 0.013; Figure 6A). As to fecal primary BAs and secondary BAs (Figure 6B), the same tendency as TBAs were found among the three groups. In the ND group, the content of fecal primary BAs and secondary BAs was 58,343 ± 25,290 and 263,960 ± 17,553, while an HFD increased the content of them (275,828 ± 149,763 and 650,898 ± 89,492, respectively; *p* = 0.017 and *p* = 0.0001, respectively). It has been observed that probiotics could decrease the concentration of both primary bile acids (124,087 ± 24,802) and secondary bile acids (464,706 ± 94,661). However, only the reduction in secondary bile acids reached statistical significance after administering probiotics (*p* = 0.017). Moreover, the concentration of fecal unconjugated BAs in HFD mice (778,273 ± 88,396) was higher than that in the ND group (305,635 ± 26,059; *p* = 0.0002). The concentration of fecal unconjugated BAs in the HFD+P group was 694,504 ± 147,345, indicating the intervention of CL probiotics decreased the upward trend of unconjugated BAs in the HFD mice, but there was no statistically significant meaning to this (*p* = 0.622; Figure 6C1). However, the concentration of fecal conjugated BAs showed a different trend among the ND group, HFD group, and HFD+P group but without statistical significance (Figure 6C2). In addition, probiotic treatment also upregulated the relative levels of secondary BAs (Figure 6D2).

HFD induced an increase in multiple unconjugated fecal BAs in mice, including ACA, DCA, HDCA, apoCA, α-MCA, β-MCA, MDCA, LCA, and UCA, which were higher in the HFD mice than in the ND group (*p* = 0.031, *p* < 0.0001, *p* = 0.017, *p* = 0.002, *p* = 0.0008, *p* = 0.004, *p* = 0.012, *p* < 0.0001, and *p* = 0.012, respectively; Figure 7A). The intervention of the CL probiotics reversed the increments of those fecal unconjugated BAs induced by HFD, but only the reduction in LCA was statistically significant (*p* = 0.016). The HFD led to a higher concentration of GLCA (17.77 ± 11.49) and a lower concentration of THCA (8.180 ± 2.168) than in the ND group (3.775 ± 0.949 and 32.68 ± 9.219, respectively; *p* = 0.041 and *p* = 0.0006, respectively). At the same time, the intervention of CL probiotics reversed the alteration of GLCA (8.365 ± 2.490) in the HFD group, but there was no statistical significance (*p* = 0.180; Figure 7B).

### Correlation analysis between fecal BA levels and gut microbiota

**Figure 8:**
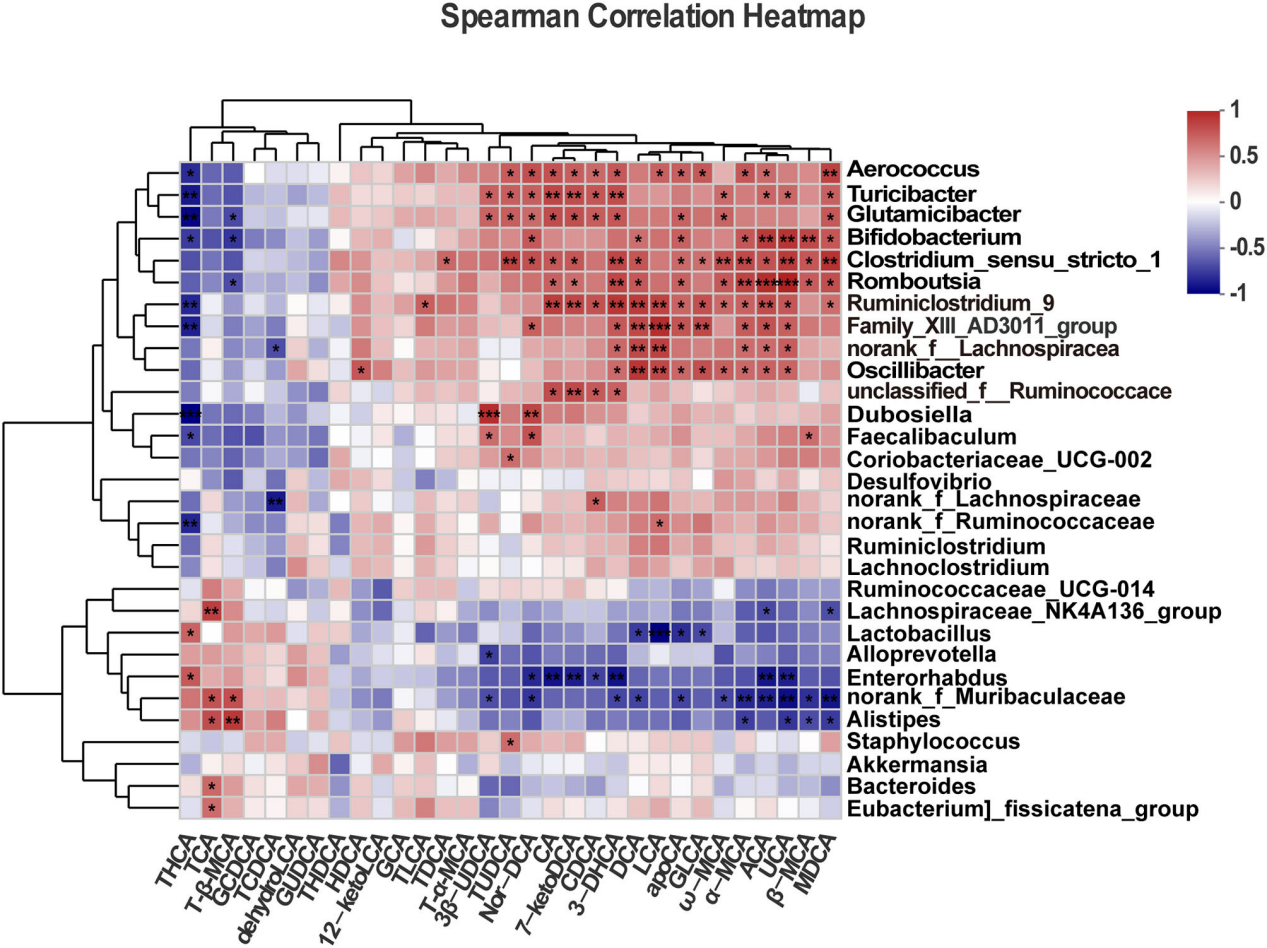
Correlation analysis between gut microbiota and fecal bile acids. All data are given as the mean ± SD (*n* = 4). *p < 0.05; **p < 0.01; ***p < 0.001.

We performed Spearman's correlation analysis to determine the association between the top 30 dominant genera and BAs. As shown in the heatmap (Figure 8), the quantity of *Bifidobacterium* in mice was positively correlated with fecal levels of Nor-DCA (*r* = 0.662, *p* = 0.019), DCA (*r* = 0.576, *p* = 0.049), apoCA (*r* = 0.633, *p* = 0.026), α-MCA (*r* = 0.669, *p* = 0.017), ACA (*r* = 0.783, *p* = 0.002), UCA (*r* = 0.804, *p* = 0.001), β-MCA (*r* = 0.733, *p* = 0.006), and MDCA (*r* = 0.669, *p* = 0.017) while showing a negative correlation with THCA (*r* = −0.640, *p* = 0.024) and T-β-MCA (*r* = −0.658, *p* = 0.019) levels. The abundance of *Enterorhabdus* was positively correlated with THCA (*r* = 0.657, *p* = 0.020) and negatively correlated with Nor-DCA (*r* = −0.706, *p* = 0.010), CA (*r* = −0.776, *p* = 0.002), 7-ketoDCA (*r* = −0.741, *p* = 0.005), CDCA (*r* = −0.622, *p* = 0.030), 3-DHCA (*r* = −0.783, *p* = 0.002), ACA (*r* = −0.769, *p* = 0.003), and UCA (*r* = −0.734, *p* = 0.006). The high level of *Bacteroides* was found to be positively correlated with fecal TCA (*r* = 0.601, *p* = 0.038). *Lactobacillus* was negatively associated with LCA (*r* = −0.832, *p* = 0.0007), apoCA (*r* = −0.685, *p* = 0.013), GLCA (*r* = −0.594, *p* = 0.041), and DCA (*r* = −0.636, *p* = 0.026) and positively correlated with THCA (*r* = 0.622, *p* = 0.030). In contrast, the genus *Ruminiclostridium-9* was positively correlated with CA (*r* = 0.769, *p* = 0.003), 7-ketoDCA (*r* = 0.734, *p* = 0.006), CDCA (*r* = −0.685, *p* = 0.013), 3-DHCA (*r* = 0.797, *p* = 0.002), DCA (*r* = 0.776, *p* = 0.003), LCA (*r* = 0.748, *p* = 0.005 < 0.01), apoCA (*r* = 0.615, *p* = 0.033), GLCA (*r* = 0.699, *p* = 0.011), ω-MCA (*r* = 0.601, *p* = 0.038), α-MCA (*r* = 0.629, *p* = 0.028), ACA (*r* = 0.713, *p* = 0.009), UCA (*r* = 0.671, *p* = 0.016), TLCA (*r* = 0.636, *p* = 0.026), and MDCA (*r* = 0.580, *p* = 0.047) and was negatively correlated with THCA (*r* = −0.713, *p* = 0.009).

## Discussion

Probiotics are known to affect the metabolism of BAs and the organization of gut microbiota (34) by regulating various molecular and cellular functions, including the FXR/FGF15 pathway (19). This pathway plays a critical role in regulating the synthesis, secretion, and circulation of BAs (35), and a certain amount of BA may be excreted from the body. Thus, confirming that the patients with NAFLD have higher levels of fecal total bile acid that correlate with gut microbiota dysbiosis and increased rates of bile acid synthesis in the liver (36). The FXR/FGF15 pathway (activated FXR agonists) can reduce the accumulation of hepatic lipids thus being used to treat NAFLD (23, 37). In the current study, we found that CL probiotics effectively recovered the hepatic pathological damage and dyslipidemia in NAFLD mice.

Hepatic steatosis and the composition of gut microbiota and bile acid metabolism were also significantly improved under the treatment of probiotics. Previously, some of the other probiotics improved the gut microbiota dysbiosis in HFD-induced NAFLD mice (38). Moreover, several probiotics (mainly *Lactobacillus* and *Bifidobacterium*) can effectively reduce hepatic lipid accumulation (38, 39) and decrease aminotransferase activity and circulating levels of pro-inflammatory mediators, such as TNF-α, IL-1β, and IL-6 in NAFLD patients (40), showing that probiotics can improve liver function and reduce inflammation to alleviate NAFLD. Based on these facts, our findings support the potential clinical use of probiotics as a safe and effective therapeutic strategy for treating NAFLD and related metabolic disorders.

Additionally, some of the probiotics, such as in HFD hyperlipidemia mice, worsened the serum lipid parameters and hepatic lipid accumulation (41) or resulted in no clinical improvement in the NAFLD patients; however, they stabilized the mucosal immune function that prevents intestinal permeability (42). On the contrary, probiotics significantly improved the lipid profile of NAFLD patients (43) and HFD mice (44). Interestingly, the probiotics we used in our study significantly reduced the levels of TC, TG, and LDL-C in the serum of NAFLD mice, and these reductions varied depending on the probiotic strain used and the duration of intervention. HDL-C has been shown as a protective factor facilitating cholesterol metabolism by transporting TC from extrahepatic tissues to the liver (45). HDL-C interacts with most peripheral cells through its receptors, including SR-B1, ATP-binding cassette transporter A1 (ABCA1), and ATP-binding cassette transporter G1 (ABCG1), which may be a critical step for facilitating HDL-to-cell communication (46). SR-B1 removes excess cholesterol from peripheral tissues and returns it to the liver for excretion (47). This process helps maintain cholesterol homeostasis confirmed by elevated levels of total cholesterol and HDL-C in SR-B1 knockout mice (48). In our study, we noticed the expression level of hepatic SR-B1 in HFD mice was lower than in the ND group, and the level of serum HDL-C was higher. Therefore, we speculated that the increased level of serum HDL-C in HFD mice may not be associated with increased production but with decreased liver intake of HDL-C. As known, the disruption of SR-B1 in the HFD mice blocks the reverse transport of TC (49, 50). Moreover, we found that probiotic intervention enhanced the expression of liver SR-B1 and slightly decreased the serum HDL-C level, indicating that the reverse transport of cholesterol and the uptake of HDL-C by the liver were recovered to some extent. The decrease in the levels of TC, TG, and LDL-C in the serum within the HFD+P group strongly suggested that the administration of probiotic supplements has a positive effect on the improvement of NAFLD in mice.

Gut microbiota is an integral player in NAFLD development, which activates an inflammatory response, increases endogenous ethanol, and impairs intestinal barrier function (13, 51). Long-term intake of a high-fat diet can significantly change the composition and abundance of gut microbiota, such as increasing Firmicutes and decreasing Bacteroidota and Actinobacteriota in NAFLD mice (52, 53). The changes in gut microbiota composition disrupt the intestinal barrier and increase intestinal permeability (51). Consistent with these studies, we observed that the probiotic treatment reduced the quantity of Firmicutes in the HFD+P group, suggesting CL probiotics promoted and enriched the diversity of gut microbiota in mice and thus improved NAFLD. Some studies have shown that HFD possibly reduces the diversity and *Lactobacillus* abundance of intestinal flora that regulate the expression of tight junction proteins, including Claudin-1 and ZO-1, and intestinal permeability (54). Our findings are consistent with previous studies that showed that HFD-feeding increases intestinal permeability by impairing tight junction protein function, such as a decrease in ZO-1 and Claudin-1 protein expression in the NAFLD (55, 56), and probiotic supplementation improves the expression of ZO-1 in the intestine (41). Our results demonstrated that the intervention of probiotics significantly upregulates the expression of Claudin-1 and Claudin-3 in the ileum in NAFLD mice, thereby improving the integrity of the mucosal barrier. However, there was no statistically significant elevation in ZO-1 expression after the intervention of CL probiotics. We extend the intervention duration to enhance the accuracy of evaluating the effect of CL probiotics. This showed that gut microbiota composition in HFD mice was destroyed, which initiated a local inflammatory response in the intestinal mucosa and increased intestinal permeability or mucosal barrier breakdown. Nevertheless, the levels of TLR4, TNF-α, and IL-1β driven by HFD in the liver were lowered by CL probiotics, suggesting the anti-inflammatory and other beneficial effects of CL probiotics.

BA metabolism is strongly associated with metabolic diseases (57), although the regulatory pathway remains unclear. Changes in the expression of enzymes and transporters involved in bile acid metabolism resulted in altered bile acid composition, with the progression of liver disease (58). Alteration in BA metabolism may have significant implications for the pathogenesis of liver diseases. BSEP is known to transport hepatic BAs from the hepatocytes into canaliculi, which helps in the absorption and excretion of BAs (22). Duan et al. (59) discovered that HFD inhibited hepatic expression levels of BSEP in mice. Our results showed significant upregulation of hepatic BSEP by probiotics, suggesting that probiotics can significantly increase BA transportation from the liver to the intestine.

Similarly, CYP7A1 is a major rate-limiting enzyme in the classic BA synthesis pathway, known to control BA metabolism through the negative feedback effect of FXR on CYP7A1 (60). Interestingly, CYP7A1 expression was recovered by *Lactobacillus* and *Pediococcu* in NAFLD mice (61). In our research, we found that HFD inhibited the expression of BSEP and activated the expression of CYP7A1, which caused disruption of BA metabolism, and the intervention of the CL probiotics recovered the expression of these factors. Therefore, we assume that the CL probiotics play a vital role as a hepatic FXR agonist that promotes BA metabolism and reduces hepatic cholesterol deposition.

The gut microbiota performs deconjugation, dehydrogenation, and isomerization of primary BAs, producing secondary BAs (62). The vast majority of bile acids are reabsorbed in the intestine, whereas the remaining portion is excreted in feces (22), which reflects the metabolism of BAs in the body (63). Studies reported that NAFLD is attenuated by probiotics by regulating the gut microbiota and BA metabolism (19). To further validate the effect of probiotics on BA metabolism in NAFLD mice, we detected the contents of BAs in the mice feces by targeted metabolomic profiles. Previously, increased levels of fecal total BAs and fecal LCA were observed in the mice with NAFLD induced by a high-fat diet (64). Our research indicated that an HFD significantly increased fecal total BAs, fecal primary and secondary BAs, fecal unconjugated BAs, and the concentration of fecal LCA. In contrast, probiotics reduced these BA levels in mice feces, showing a consistent trend of serum cholesterol. Moreover, we noticed a significant negative correlation between the fecal LCA content and *Lactobacillus*, which may regulate BA metabolism. The changes in the composition of gut microbiota alter the content of fecal bile acids (65). Mice fecal BA metabolomics data revealed that HFD significantly increased BA synthesis (66), resulting in an elevated fecal excretion of bile acids. However, when probiotics were administered, they could recover the bile acid balance by regulating their content and composition in our study. We indicated that probiotic intervention could affect the composition of gut microbiota, which directly affects the levels and composition of BAs in NAFLD mice.

FGF19 in humans and FGF15 in mice are induced by the activation of FXR in the ileum (23, 35, 67), and probiotics may affect bile acid metabolism by upregulating the expression of the FXR-FGF15 pathway (19). Additionally, we analyzed the expression levels of FGFR4, SHP, and CYP7A1 to gain further insights into how probiotics improve bile acid metabolism. Owing to the presence of a portal vein, intestinal FGF15 can enter the liver and bind to the receptor FGFR4, thereby inhibiting the expression of CYP7A1 (68). We found that, in the liver, a high-fat diet attenuated the expression of SHP and FGFR4 and enhanced the expression of CYP7A1. Similarly, an increase in serum level of total bile acid in HFD FXR^−/−^ mice was observed. After probiotic intervention, the SHP protein expression level did not change, while the expression level of FGFR4 and FGF15 protein was upregulated, and the expression level of CYP7A1 protein was downregulated. These findings suggest that probiotics regulated the intestinal FXR-FGF15 pathway to improve NAFLD in mice but do not affect the hepatic FXR-SHP pathway.

Our study has some limitations such as no wild-type (WT) mice were used as the negative control because previously, after probiotic intervention, we did not find any statistical significance in the diversity of gut microbiota and gut bacterial composition (data not shown). Moreover, owing to time limitations, we could not continue screening for additional probiotics to investigate whether different types of probiotics have varying mechanisms for improving NAFLD. Therefore, we recommend scrutinizing various probiotic strains to study their roles in NAFLD.

## Conclusion

In conclusion, our study suggested that CL probiotics exhibit a preventive and promising role in improving NAFLD by regulating BA metabolism and gut microbiota composition. The probiotic intervention can improve liver function and lipid deposition in mice, reducing an increase in body weight of mice. In parallel, CL probiotics, which act as selective intestinal FXR agonists, may improve BA metabolism and transport by regulating the FXR-FGF15 pathway, thereby alleviating NAFLD in high-fat diet FXR^−/−^ mice.

## Data availability statement

The original contributions presented in the study are included in the article, and the raw sequencing data are deposited in the NCBI Sequence Read Archive (SRA) accession number (https://www.ncbi.nlm.nih.gov/bioproject/PRJNA933781).

## Ethics statement

The animal study was reviewed and approved by the Animal Ethics Committee of the Fifth Affiliated Hospital of Zhengzhou University (KY2021039).

## Author contributions

MY performed the experiments and wrote the manuscript. IB reviewed and revised this manuscript. HW and YZ analyzed the data and wrote the manuscript. HH and XS performed the experiments. YY and YM contributed to review and editing. LM and PZ conceived and designed the experiments and revised the manuscript. All authors contributed to the article and approved the submitted version.
