# Supplementary material for: Effects of cholesterol-lowering probiotics on non-alcoholic fatty liver disease in FXR gene knockout mice
Source: Front Nutr. 2023 Jul 20;10:1121203. doi: 10.3389/fnut.2023.1121203 (PMC10397539; doi:10.3389/fnut.2023.1121203)
Supplement: Supplementary file 1 [file Image_1.pdf]

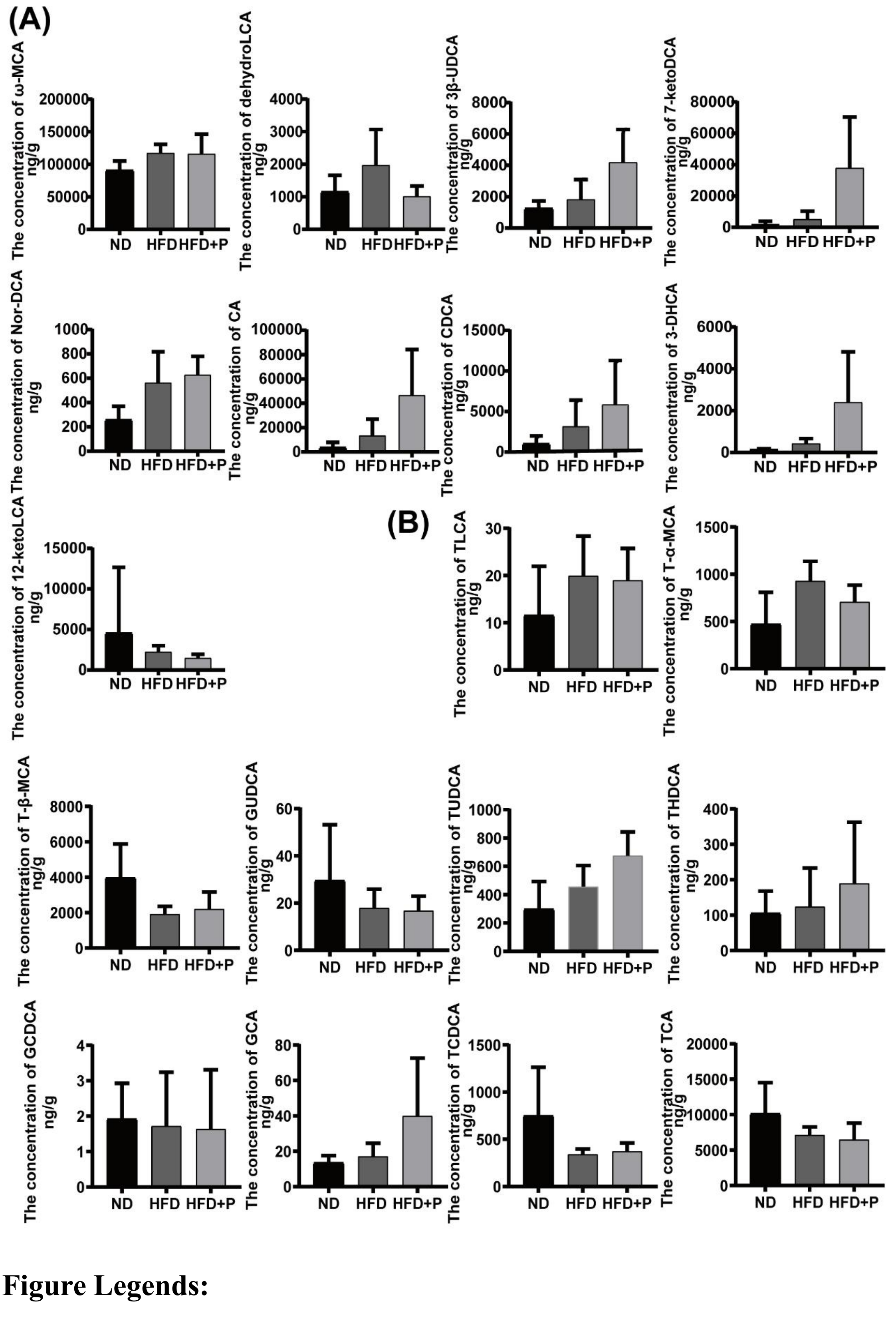

Figure Legends:

Supplement Figure1. Effect of cholesterol-lowering probiotics on fecal bile acids monomers. (A) unconjugated bile acids monomers; (B) conjugated bile acids

monomers. ω-MCA, ω-muricholic acid; dehydroLCA, dehydrolithocholic acid; 3-DHCA, 3-dehydrocholic acid; 3β-UDCA, 3β-ursodeoxycholic acid; 7-ketoLCA, 7-ketolithocholic acid; 3β-UDCA, 3β-ursodeoxycholic acid; 7-ketoLCA, 7-ketolithocholic acid; Nor-DCA, norcholic acid; CA, cholic acid; CDCA, chenodeoxycholic acid; 12-ketoLCA, 12-ketolithocholic acid; GCDCA, glycochenodeoxycholic acid; GCA, glycocholic acid; TCDCA, taurochenodeoxycholic acid; TCA, taurocholic acid; GUDCA, glycoursodeoxycholic acid; THDCA, taurohyodeoxycholic acid; TUDCA, tauroursodeoxycholic acid; TLCA, taurolithocholic acid; T-α-MCA, tauro-α-muricholic acid; T-β-MCA, tauro-β-muricholic acid. All data are given as the mean ± SD ($n=4$). *$p < 0.05$; **$p < 0.01$

ND, normal diet; HFD, high-fat diet; HFD+P, probiotics intervention in HFD
